# Signs and symptoms of acromegaly at diagnosis: the physician’s and the patient’s perspectives in the ACRO-POLIS study

**DOI:** 10.1007/s12020-018-1764-4

**Published:** 2018-09-29

**Authors:** Philippe Caron, Thierry Brue, Gérald Raverot, Antoine Tabarin, Anne Cailleux, Brigitte Delemer, Peggy Pierre Renoult, Aude Houchard, Fatine Elaraki, Philippe Chanson

**Affiliations:** 1 Hôpital Larrey, Toulouse, France; 2 Aix-Marseille University, INSERM, MMG, AP-HM, Hôpital de la Conception, CRMR HYPO, Marseille, France; 3 Groupement Hospitalier Est, Lyon, France; 4 Hôpital Haut Lévêque-CHU de Bordeaux, Bordeaux, France; 5 Rouen University Hospital, Endocrinology Unit, Inserm CIC-CRB 1404, F 76 000 Rouen, France; 6 CHU de Reims, Reims, France; 7 CHU Bretonneau, Tours, France; 8 Ipsen Pharma, Boulogne-Billancourt, France; 9 Assistance Publique-Hôpitaux de Paris, Hôpital Bicêtre, Centre de Référence des Maladies Rares de l’Hypophyse HYPO, F94275 Le Kremlin-Bicêtre, France; 10 Université Paris-Sud, Le Kremlin-Bicêtre, France

**Keywords:** Acromegaly, Multiple correspondence analysis, Sign-and-symptom association, Diagnosis

## Abstract

**Purpose:**

Acromegaly is characterized by a broad range of manifestations. Early diagnosis is key to treatment success, but is often delayed as symptomatology overlaps with common disorders. We investigated sign-and-symptom associations, demographics, and clinical characteristics at acromegaly diagnosis.

**Methods:**

Observational, cross-sectional, multicenter non-interventional study conducted at 25 hospital departments in France that treat acromegaly (ClinicalTrials.gov: NCT02012127). Adults diagnosed with acromegaly < 5 years were enrolled. Demographic and clinical data were obtained from medical reports and patient questionnaires. Sign-and-symptom associations were assessed by multiple correspondence analysis (MCA).

**Results:**

Overall, 472 patients were included in the analyses. MCA was unsuccessful in identifying sign-and-symptom associations at diagnosis. Endocrinologists (29.5% patients) and other clinical specialists (37.2% patients) were commonly first to suspect acromegaly. Morphologic manifestations (83.7–87.9% patients), snoring syndrome (81.4% patients), and asthenia (79.2% patients) were frequently present at diagnosis; differences were found between sexes for specific manifestations. Rates of discrepancy between patient- and physician-reported manifestations were highest for functional signs. Earliest manifestations prior to diagnosis, according to how they were detected, were enlarged hands and feet (6.4 ± 6.8 and 6.2 ± 6.9 years, functional signs), hypertension (6.6 ± 7.5 years, complementary examination) and carpal/cubital tunnel syndrome (5.7 ± 6.7 years, functional signs with complementary examination).

**Conclusions:**

Results confirm the broad range of manifestations at diagnosis and delay in recognizing the disease. We identified early manifestations and sex differences that may aid physicians in diagnosing acromegaly. Discrepancy rates suggest physicians should obtain the patient’s perspective and seek functional signs during diagnosis.

## Introduction

Acromegaly is a rare (incidence of 3.3 per million per year) [1], chronic, multisystem disease characterized by excessive growth hormone (GH) secretion and elevated insulin-like growth factor-1 (IGF-1) levels. Acromegaly, often caused by a benign pituitary adenoma, manifests as a broad range of signs, symptoms, and comorbidities caused by the tumor (headaches and visual field defects) and by the long-term effects of GH/IGF-1 hypersecretion on multiple organs and tissues. Typical manifestations include morphologic changes (broadening of hands, feet and facial features, prognathism, frontal bossing, and dorsal kyphosis with deformation of the rib cage in severe cases), cardiovascular disorders, osteoarticular and metabolic manifestations, sleep apnea, respiratory disease, neuropathies, sexual disorders, and gastrointestinal manifestations) [2, 3]. The severity of clinical manifestations depends on the levels of GH and IGF-1, tumor size, and time to diagnosis.

Treatment options for acromegaly include surgery, medical therapy, and radiotherapy, which aim to control clinical signs and symptoms, normalize GH/IGF-1 excess, and reduce or remove the tumor mass [4]. Early diagnosis is considered to be a critical factor in the rate of treatment success and is important for preventing long-term comorbidity and premature death [5–8]. However, the diagnosis of acromegaly is often delayed, and has been reported to be up to 20 years [9–13]. More recent data suggest that the delay to acromegaly diagnosis is now reduced to 3–6 years, a reduction that may be attributed to improved GH and IGF-1 assays, increased use of magnetic resonance imaging for the assessment of head-related complaints, increased acromegaly awareness among endocrinologists, and the increased availability of information to patients in the internet era [14–17]. Despite these improvements, difficulties in diagnosis still exist due to the insidious onset of clinical features, overlap of these with other common conditions, and lack of disease awareness among other medical specialists. Indeed, due to the multisystem nature of the disease, patients may visit a number of different medical specialists to be treated for individual manifestations before the possibility of acromegaly is first suggested [4, 16, 18]. Therefore, improving physician awareness of the signs, symptoms, and comorbidities of acromegaly remains key to early diagnosis [19].

The purpose of the ACRO-POLIS study was to identify features that would aid physicians in earlier identification of the disease, and further reduce the delay to diagnosis and treatment. Here, we describe at diagnosis the demographic and clinical characteristics of a large cohort of patients with acromegaly. We show the frequency of signs, symptoms, and comorbidities at diagnosis, together with attempts to identify characteristic sign-and-symptom associations. In addition, we describe the occurrence of signs, symptoms, and comorbidities in the years prior to diagnosis and we report differences between men and women in their manifestations; and—for the first time—discrepancies between patient and physician-reported manifestations of acromegaly.

## Subjects and methods

### Study design and patients

This non-interventional, epidemiological, observational, multicenter, cross-sectional study was conducted by endocrinologists at 25 hospital departments in France known to treat and follow-up patients with acromegaly. Information about the signs, symptoms, and comorbidities that were present at acromegaly diagnosis were collected between 2013 and 2014. Participation was proposed to 62 endocrinologists known to treat acromegaly on the basis of their involvement in the French Acromegaly Registry [20] and/or the Club Français de l’Hypophyse (the study group of the French Endocrine Society on pituitary disorders). Endocrinologists were required to recruit at least one patient to be considered as active in the study. It was expected that 20 endocrinologists would participate in the study. Patients (≥18 years old) diagnosed with acromegaly for less than 5 years previously were included; patients who objected to the collection of their data were excluded. Participating endocrinologists were asked to screen all patients meeting the inclusion criterion, and send them an information sheet and patient questionnaire.

Before study initiation, written and dated approval or favorable opinion was obtained from the independent ethics committee or institutional review board. In France, only interventional studies need to be submitted to a Committee for the Protection of the Persons (CPP). No submission to a CPP was therefore required for this non-interventional study. Informed consent was obtained from all individual participants included in the study prior to enrollment, and the study was conducted in accordance with the Declaration of Helsinki, Good Epidemiology Practice, and local regulatory requirements applicable to non-interventional studies. The study is registered at ClinicalTrials.gov (NCT02012127).

### Data collection and study endpoints

Data were collected retrospectively from patients’ medical records (transcribed into case report forms [CRFs]; Online Resource 1), and from questionnaires written in lay terms and completed by patients (Online Resource 2). Demographic and disease data were captured, including the signs, symptoms, and comorbidities of acromegaly at diagnosis and the dates of their first occurrence.

The study endpoints were to describe the most characteristic sign-and-symptom associations present at the diagnosis of acromegaly (primary endpoint), and to describe demographic and clinical characteristics at the diagnosis of acromegaly (secondary endpoint). Post hoc analyses were conducted to: assess the timing of the occurrence of manifestations in the years prior to diagnosis; report differences in manifestations between men and women; and to examine discrepancies between data captured in the CRF by the physician versus the patient questionnaire.

### Statistical methods

#### Sample size

The sample size was based on the number of patients required to ensure the reliability of the primary-endpoint analysis (multiple correspondence analysis, MCA). Considerations were: including ten patients per factor analyzed [21] or 500 patients overall [22], or adopting a 20:1 ratio of patients to number of factors analyzed [23]. A sample size of 500 patients would ensure very robust results for the analysis of up to 40 factors according to these criteria. The sample size was increased to 550 to allow for 10% of patients having missing data.

#### Multiple correspondence analysis (MCA)

MCA is a powerful analytic technique used to detect and represent the pattern of relationships of variables and explore underlying structures in large, complex datasets containing categorical data. MCA has been used been used previously to identify sign-and-symptom associations from clinical datasets [24, 25]. MCA describes relationships between categorical variables within a dataset and represents the frequency of each variable in terms of the distance between individual variables, and the distance to the average variable profile (explained by the level of inertia), as a cloud of points in a two-dimensional map [26]. Factorial axes are derived in order to identify which variables differ the most between patients, enabling the differentiation of patient profiles [26]. An a priori MCA was undertaken to evaluate sign-and-symptom associations at diagnosis of acromegaly (primary endpoint), using factors (e.g., morphologic manifestations) and associated variables (e.g., facial modifications, prognathism) derived from CRFs and patient questionnaires. Inconsistencies between CRFs and patient questionnaires were managed according to the scheme in Online Resource 3. Two types of variable were used: active variables were used to construct axes; supplementary variables were projected onto the dimensions of the original result to aid interpretation only. The a priori MCA (primary endpoint) was conducted with 38 active and 16 supplementary variables, and data from 319 patients. To investigate whether the quality of representation could be improved by increasing the number of patients with data and decreasing the number of variables, a post hoc MCA was subsequently conducted which omitted six factors (BMI, educational level, employment status, first person who suspected acromegaly, type of tumor, size of tumor), grouped manifestations into fewer variables overall (e.g., rachialgia and arthropathy combined into osteoarticular manifestations), and excluded morphologic manifestations entirely. This MCA comprised 19 active and two supplementary variables and data from 405 patients.

#### Discrepancies between manifestations reported by patients and physicians

A rate of discrepancy was calculated for differences between a patient’s answer in the questionnaire and the corresponding information in the CRF. A discrepancy was apparent if “No” was ticked in the patient questionnaire and “Yes” was ticked in the CRF, or vice versa.

#### Categorization of manifestations according to how they were detected (post hoc analysis)

Manifestations were categorized into three groups according to how they were detected. Specifically, functional signs (FS; were those detected after patients report the manifestation, or after clinical examination), comorbidities diagnosed based on complementary examinations (CE), and symptoms or comorbidities diagnosed as FS and then confirmed by complementary examinations (FS + CE). These categories were adopted to give an insight into the occurrence of manifestations prior to diagnosis, and any discrepancies between manifestations reported by patients and physicians.

#### Statistical analyses

Analyses were conducted using the analysis population (patients with both a complete CRF and patient questionnaire). Statistical analyses were carried out using SAS software version 9.2. Unless stated otherwise, results are expressed as means, standard deviations (SDs), and 95% confidence intervals (CIs).

## Results

### Patient disposition and baseline characteristics

An unexpectedly large proportion of the endocrinologists contacted responded (52/62) and then agreed to recruit at least one patient (37/62). As one physician did not recruit any patients, the total number of participating physicians was 36. Of the 16 physicians who responded but did not participate: five physicians indicated that they did not usually participate in a study; three stated a lack of interest; two stated a lack of time; three agreed to participate but either did not initiate a visit or recruit a patient; two withdrew from the study before sending the patient questionnaire; and data were missing for one physician. Participating and non-participating endocrinologists were representative of the different areas of France. In total, 648 patients were included in the study (global population), which was greater than the planned sample size of 550 patients. A total of 176 patients from the global population (*n* = 648) were excluded due to protocol deviations: 142 patients had a missing patient questionnaire, 39 patients had a diagnosis of acromegaly greater than 5 years before inclusion, 5 patients had at least one missing eligibility criterion (some patients had more than one reason for exclusion). Analyses were conducted on the remaining 472 patients (analysis population), who had both a completed CRF and patient questionnaire. Baseline demographic and disease characteristics are shown in Table 1. At baseline, mean (±SD) age was 51.9 (±14.3) years, 42.8% of patients were men, with a microadenoma (19.5%) or macroadenoma (80.5%).

**Table 1:** Baseline demographic and disease characteristics (analysis population)

	Analysis population (*n* = 472)
Age (years)	51.9 (±14.3)
BMI (kg/m^2^)	*n* = 436
	27.7 (±5.3)
Sex, *n* (%)	
Men	202 (42.8)
Women	270 (57.2)
Time since diagnosis (months)	30.6 (±17.8)
Acromegaly first suspected by, *n* (%):	*n* = 427
Endocrinologist	126 (29.5)
General practitioner^a^	69 (16.2)
Other specialist	159 (37.2)
Others^b^	73 (17.1)
Type of pituitary adenoma	*n* = 462
GH	364 (78.8)
GH/prolactin	84 (18.2)
Other	14 (3.0)
Tumor size	*n* = 456
Microadenoma	89 (19.5)
Macroadenoma	367 (80.5)
GH (ng/mL)	*n* = 277
≤2.5	48 (17.3)
>2.5	229 (82.7)
IGF-1 (% ULN)	*n* = 406
<100	7 (1.7)
100–130	20 (4.9)
>130	379 (93.3)
Serum prolactin^c^ (µg/L)	*n* = 62
	183 (±650)

Data are mean (±SD) unless stated otherwise from the analysis population (patients with both a complete CRF and patient questionnaire)

^a^General practitioner (13.8%) and general practitioner equivalent (2.3%)

^b^healthcare professional (7.3%), patient (3.7%), patients’ relatives (3.3%) and other (2.8%)

^c^for GH and prolactin adenomas. *BMI* body mass index, *CRF* case report form, *GH* growth hormone, *IGF-1* insulin-like growth factor-1, *SD* standard deviation, *ULN* upper limit of normal

### Sign-and-symptom associations at diagnosis

As a result of missing data, the a priori MCA included data from only 319 patients from the analysis population (*n* = 472). The first three axes of the a priori MCA explained only 19.0% of the total inertia (Table 2) and did not allow the identification of characteristic sign-and-symptom associations at diagnosis. The primary endpoint was therefore considered to be negative and because of this, the a priori MCA maps are not reported here. A post hoc MCA was undertaken in an attempt to improve the quality of representation. In this MCA, the numbers of factors and the categories of individual variables were reduced, morphologic manifestations were removed, and data included from a greater number of patients (*n* = 405, analysis population). Despite these measures, the first three axes of the post hoc MCA explained only 25.1% of the total inertia (Table 2). Therefore, the maps for the post hoc MCA are also not reported here. Given the large ACRO-POLIS dataset of demographic and clinical characteristics in patients with acromegaly, this report will instead focus on the secondary endpoint and other post hoc analyses to identify features that may aid physicians in their diagnosis of acromegaly.

**Table 2:** Inertia decomposition of the MCA

A priori MCA (*N* = 319 patients)^a^		Post hoc MCA (*N* = 405 patients)^a^	
Percentage of inertia	Cumulative percentage of inertia	Percentage of inertia	Cumulative percentage of inertia
8.03 (axis 1)	8.03	10.56 (axis 1)	10.56
5.86 (axis 2)	13.89	7.69 (axis 2)	18.25
5.12 (axis 3)	19.01	6.84 (axis 3)	25.09

^a^Analysis population. *MCA* multiple correspondence analysis

### Manifestations at diagnosis

#### Frequency

**Fig. 1:**
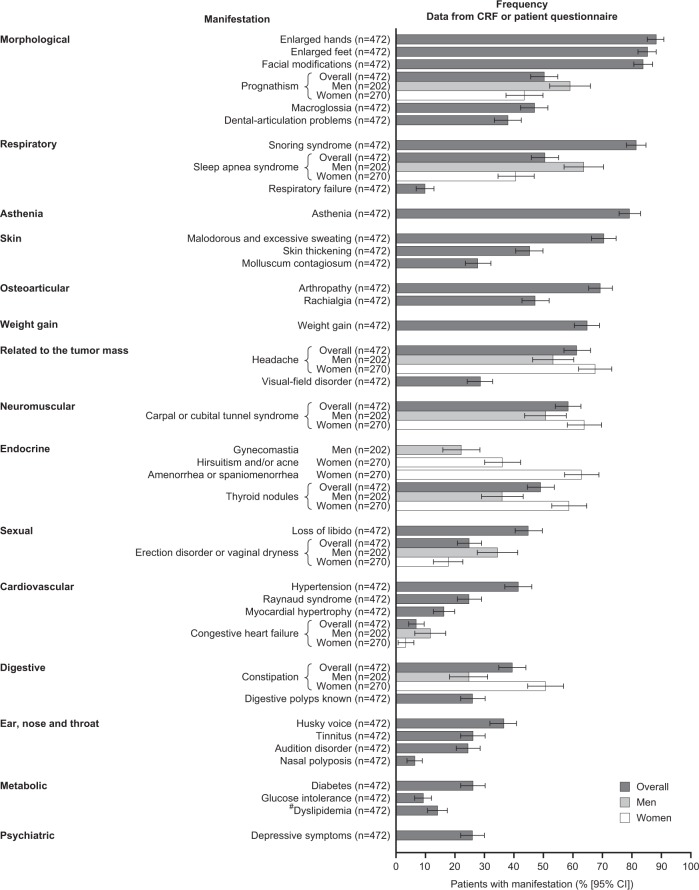
Frequency of symptoms and comorbidities at diagnosis of acromegaly (analysis population; secondary endpoint) with sex differences. Bars represent the percentage of patients with each manifestation. Error bars represent 95% CI. *CI* confidence intervals, *CRF* case report form. ^#^Re-classified manifestation

At diagnosis, patients presented a broad range of signs, symptoms, and comorbidities (Fig. 1). The most frequent manifestations were morphologic (enlarged hands, enlarged feet), facial modifications (frontal bump, enlargement of the nose), snoring syndrome, and asthenia. The manifestations that were more common in women than men were headache, carpal or cubital tunnel syndrome, constipation, and thyroid nodules. Other manifestations more common in men than women were prognathism, sleep apnea syndrome, congestive heart failure, and erection disorders (versus vaginal dryness in women).

#### Discrepancies between the patient questionnaire and the CRF (post hoc analysis)

**Fig. 2:**
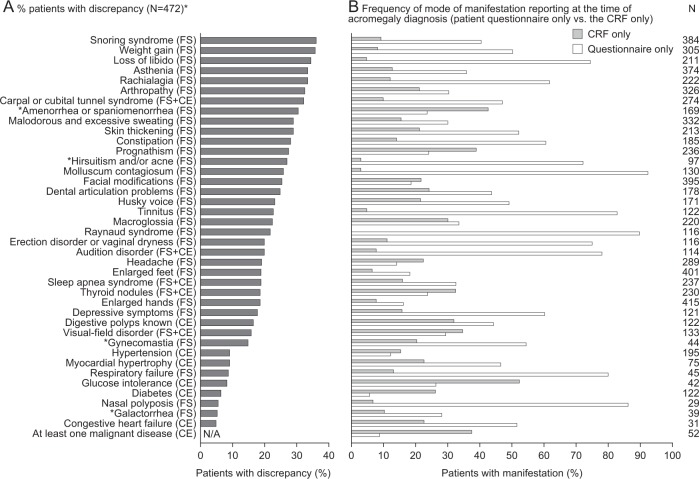
**a** Discrepancies between manifestations reported in the CRF versus the patient questionnaire at time of acromegaly diagnosis (analysis population; post hoc analysis), **b** frequency of mode of manifestation reporting at acromegaly diagnosis (patient questionnaire only versus the CRF only) (analysis population). *N = 472 except amenorrhea or spaniomenorrhea *n* = 269; galactorrhea *n* = 270; gynecomastia *n* = 202; hirsutism and/or acne *n* = 270. ^†^Amenorrhea or spaniomenorrhea, galactorrhea; and hirsutism and/or acne are displayed as % of women; gynecomastia is displayed as % of men. For % patients with discrepancy, a discrepancy is defined as a sign or comorbidity reported in either the CRF or patient questionnaire, but not both. *CE* comorbidities diagnosed based on complementary examinations; *CRF* case report form, *FS* functional signs (detected after patients report the manifestation, or after clinical examination); FS + CE, symptoms or comorbidities diagnosed on functional signs and confirmed by complementary examinations

Of the 39 manifestations reported, rates of discrepancy ranged from 5.5–36.2%. Manifestations with the highest rates were predominantly FS: snoring, weight gain, loss of libido, asthenia, rachialgia, and arthropathy (Fig. 2a). Lowest rates of discrepancy were observed in congestive heart failure (CE), galactorrhea (FS), nasal polyposis (FS), diabetes (CE), glucose intolerance (CE), and respiratory failure (FS). Overall, manifestations were reported more frequently at the time of acromegaly diagnosis in the patient questionnaire only, than in the CRF only (Fig. 2b). This was most apparent for loss of libido (FS), hirsutism and/or acne (FS), molluscum contagiosum (FS), tinnitus (FS), Raynaud syndrome (FS), erection disorder or vaginal dryness (FS); audition disorder (FS + CE), respiratory failure (CE), and nasal polyposis (FS).

### Manifestations prior to diagnosis (post hoc analysis)

#### Frequency at timeframes prior to diagnosis

**Fig. 3:**
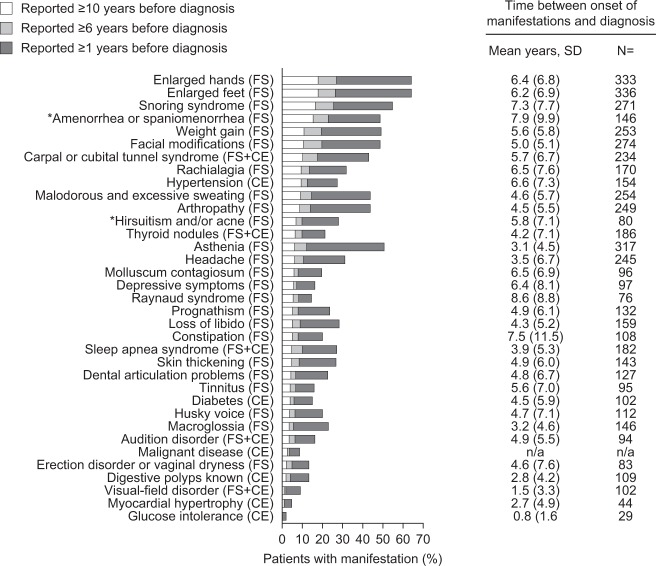
Occurrence of manifestations at timeframes prior to diagnosis (analysis population; post hoc analysis) reported by patients. *Amenorrhea or spaniomenorrhea, galactorrhea; and hirsutism increase and/or acne are displayed as % of female patients only gynecomastia are displayed as % of male patients only). Signs, symptoms and comorbidities prelisted in the CRF and in the patient questionnaire that were reported by patients are described at timeframes before the diagnosis of acromegaly. Results for frequency of manifestations at timeframes prior to diagnosis are *n* = 472 except for amenorrhea or spaniomenorrhea, galactorrhea, hirsutism increase and/or acne (*n* = 270); and gynecomastia (*n* = 202). *CE* comorbidities diagnosed based on complementary examinations, *CRF* case report form, *FS* functional signs (detected after patients report the manifestation, or after clinical examination); FS + CE, symptoms or comorbidities diagnosed on functional signs and confirmed by complementary examinations; *SD* standard deviation

The most frequent manifestations at timeframes prior to diagnosis are shown in Fig. 3. The most frequent FS were enlarged hands (18.2% at ≥10 years, 26.9% at ≥6 years, and 62.9% at ≥1 year prior to diagnosis) and enlarged feet (18.2% at ≥10 years, 26.3% at ≥6 years, and 62.9% at ≥1 year prior to diagnosis). The most frequent CE was hypertension (9.7% at ≥10 years, 12.7% at ≥6 years, and 26.9% at ≥1 year prior to diagnosis). The most frequent FS + CE manifestation was carpal or cubital tunnel syndrome (10.4% at ≥10 years, 17.6% at ≥6 years, and 42.2% at ≥1 year prior to diagnosis).

#### Time from onset to diagnosis

The delay between manifestation onset and diagnosis was considered at two levels: for each patient (from the onset of the first manifestation); and for each individual manifestation (all patients reporting the individual manifestation). The mean (±SD) time between the onset and diagnosis for each patient (analysis population) was 14.2 (±11.3) years (*n* = 469; 95% CI: 13.1, 15.2) (post hoc analysis). The mean (±SD) time between onset and diagnosis for each individual manifestation is shown in Fig. 3. FS apparent earlier in the disease course included morphologic manifestations (enlarged hands, enlarged feet, and facial modifications), snoring, and weight gain. The CE manifestation present prior to diagnosis was hypertension, while the earliest occurring FS + CE manifestation was carpal or cubital tunnel syndrome.

**Fig. 4:**
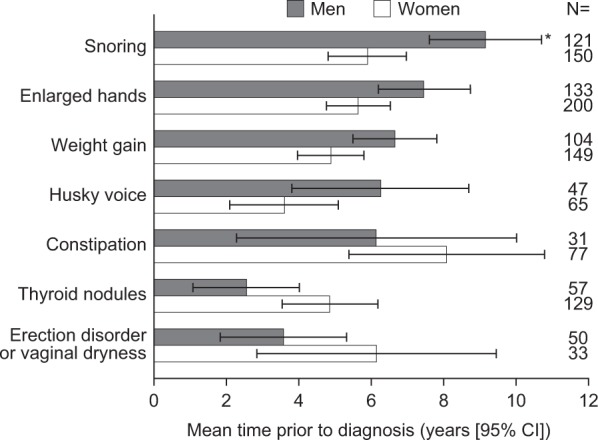
Mean time prior to diagnosis (years) between the detection of early acromegaly manifestations and diagnosis in men and women (analysis population; post hoc analysis). Bars represent the mean number of years prior to acromegaly diagnosis. Error bars represent 95% CI. *Represents statistical significance vs. women. *CI* confidence intervals

There were differences between men and women in the occurrence of a number of manifestations (Fig. 4). Manifestations detected earlier in men, compared with women, that may be clinically relevant include enlarged hands, weight gain, and husky voice. Conversely, manifestations that were detected earlier in women than in men that may have clinical relevance include thyroid nodules.

## Discussion

The purpose of these analyses was to identify features that would aid physicians in their earlier identification of the disease, and further reduce the delay to diagnosis and treatment. MCA did not allow identification of any sign-and-symptom associations at diagnosis; however, the secondary endpoint and other analyses revealed a number of clinically relevant findings that may help physicians in their diagnosis, including: the most frequent manifestations at diagnosis; discrepancy rates between patient- and physician-reported manifestations; and differences between men and women in the frequency and onset of manifestations.

Although using MCA to identify sign-and-symptom associations at diagnosis was unsuccessful in these analyses, a recent case-control study found strong associations between manifestations and diagnosis using univariate and multivariate regression models, despite the inclusion of fewer patients [27]. Nevertheless, our study highlights the difficulty in identifying manifestations, and clusters of these, that would raise acromegaly awareness in physicians and support clinical screening for early acromegaly diagnosis.

Among the findings that may help physicians, the manifestations more frequently presented at diagnosis were morphologic, as well as snoring syndrome and asthenia. Meanwhile, enlarged hands and feet (FS), hypertension (CE), and carpal or cubital tunnel syndrome (FS + CE) were identified as the most frequent manifestations within the FS, CE and FS + CE categories at ≥10 years, ≥6 years, and ≥1 year prior to diagnosis (post hoc analysis). A novel and clinically important finding from the ACRO-POLIS study was the identification of discrepancies between the reporting of manifestations by the patient and physician (post hoc analysis). Rates of discrepancy at diagnosis were highest for a number of FSs, which tended to be under-reported in the CRF, a trend that was also apparent among the reporting of a number manifestations occurring earliest in the years before diagnosis (snoring syndrome [FS], weight gain [FS], enlarged hands [FS], enlarged feet [FS], and carpal/cubital tunnel syndrome [FS + CE]). The causes of reporting discrepancies are likely to be manifold. Symptoms of acromegaly often overlap with common disorders, there is potential for them to go unnoticed due to a slow onset, and there may be patient denial and/or a reluctance to report certain manifestations [18]. Symptoms may have been additionally dismissed by physicians frequently visited by patients with disparate manifestations of undiagnosed acromegaly [18, 28]. Discrepancies between patient- and physician-reported manifestations may also be explained, in part, by a lack of familiarity among patients with the medical terminology used in consultations and/or relating that terminology to the lay terms used in the patient questionnaire. In general, however, manifestations based on CE (such as glucose intolerance, diabetes, and congestive heart failure) were less likely to have discrepancies in reporting between the patient and physician. Overall, the ACRO-POLIS study highlights that during consultations, physicians should encourage patients to report all symptoms, even if believed by the patient to be unrelated or embarrassing, with an emphasis on FS. Physicians may also be encouraged to consider these symptoms collectively. In doing so, physicians are likely to assemble a more complete clinical picture that would help further reduce the delay to diagnosis and treatment.

This cross-sectional observational study was not without limitations. Aspects of the study that may have influenced key findings include the reliance on patient recall. ACRO-POLIS was designed to mitigate recall bias by excluding patients with a time since diagnosis of >5 years; combining data collected from patient medical records in a CRF with data from patient questionnaires also acted to minimize recall bias. Collection of data from patients’ medical records may have been susceptible to bias given the subjective nature of the manifestations reported. This concern was mitigated, in part, through the use of clinical research associates, rather than endocrinologists, to complete each CRF from patients’ medical records. Missing data may have affected the robustness and reliability of the MCA. To mitigate such concerns, variables for which the observation was missing in >15% of patients were excluded. The a priori MCA was conducted with fewer than the ten patients per variable recommended by Everitt [21]. While this may have made it more difficult to determine sign-and-symptom associations, associations were also not apparent in the post hoc MCA. The latter analysis included fewer factors, fewer categories for individual variables, omitted morphologic manifestations, and included data from a greater number of patients. Finally, ACRO-POLIS was conducted at sites in France and represents diagnosis of acromegaly in the French healthcare system.

Despite these limitations, data from the ACRO-POLIS study generally resonate with, and expand upon, those from other published studies. There is concordance with respect to baseline characteristics, including the frequencies of microadenoma and macroadenoma, [1, 14–16, 27, 29, 30] and the importance of endocrinologists and general practitioners in the diagnosis of acromegaly [14–16]. Our study also revealed that a range of other clinical specialists frequently were the first to suspect acromegaly, reflecting the multisystem nature of the condition. The finding that hand, foot, and facial modifications were the most frequent manifestations at diagnosis is consistent with a number of studies [1, 16]. However, these studies also reported a high prevalence of sweating: in our population, snoring syndrome and asthenia were more frequent than sweating [1, 16]. Our observation that the frequency of certain manifestations differed between men and women accords with reports of differences in GH and IGF-1 levels and subsequent acromegaly diagnosis in men and women [15, 31]. The measures of the delay to diagnosis generally accord with previous studies (3–20 years) [9–16]. The observed trend in recent years towards a general reduction in the delay in acromegaly diagnosis may play an important role in the treatment of excessive GH/IGF-1 levels and their associated comorbidities, thus reducing morbidity and mortality in acromegaly.

## Conclusions

The ACRO-POLIS study provides real-world insights into the frequency of signs, symptoms, and comorbidities at acromegaly diagnosis, and their occurrence in the years preceding diagnosis in an effort to further improve early detection of the disease. Our study emphasizes the importance of both the physicians’ awareness of acromegaly and capturing patient-reported symptoms during the consultation.

## Supplementary information

Supplementary Information
