# Correction to: Signs and symptoms of acromegaly at diagnosis: the physician’s and the patient’s perspectives in the ACRO-POLIS study

**DOI:** 10.1007/s12020-018-1789-8

**Published:** 2018-10-31

**Authors:** Philippe Caron, Thierry Brue, Gérald Raverot, Antoine Tabarin, Anne Cailleux, Brigitte Delemer, Peggy Pierre Renoult, Aude Houchard, Fatine Elaraki, Philippe Chanson

**Affiliations:** 1 Hôpital Larrey, Toulouse, France; 2 Aix-Marseille University, INSERM, MMG, AP-HM, Hôpital de la Conception, CRMR HYPO, Marseille, France; 3 Groupement Hospitalier Est, Lyon, France; 4 Hôpital Haut Lévêque-CHU de Bordeaux, Bordeaux, France; 5 Rouen University Hospital, Endocrinology Unit, Inserm CIC-CRB 1404, F 76 000 Rouen, France; 6 CHU de Reims, Reims, France; 7 CHU Bretonneau, Tours, France; 8 Ipsen Pharma, Boulogne-Billancourt, France; 9 Assistance Publique-Hôpitaux de Paris, Hôpital Bicêtre, Centre de Référence des Maladies Rares de l’Hypophyse HYPO, F94275 Le Kremlin-Bicêtre, France; 10 Université Paris-Sud, Le Kremlin-Bicêtre, France

**Correction to: Endocrine** 10.1007/s12020-018-1764-4

The original version of this article unfortunately contained a mistake in corresponding author name as Philippe Chanson in the affiliation section.

The correct corresponding author name is Philippe Caron.

