# Supplementary material for: Signs and symptoms of acromegaly at diagnosis: the physician’s and the patient’s perspectives in the ACRO-POLIS study
Source: Endocrine. 2018 Sep 29;63(1):120–9. doi: 10.1007/s12020-018-1764-4 (PMC6329724; doi:10.1007/s12020-018-1764-4)
Supplement: Supplementary file 1 — Supplementary Information [file 12020_2018_1764_MOESM1_ESM.docx]

# Signs and symptoms of acromegaly at diagnosis: The physician’s and the patient’s perspectives in the ACRO-POLIS study

# Supplementary material – *Endocrine*

Philippe Caron, Thierry Brue, Gérald Raverot, Antoine Tabarin, Anne Cailleux, Brigitte Delemer, Peggy Pierre Renoult, Aude Houchard, Fatine Elaraki, Philippe Chanson

**Corresponding author:** Professor Philippe Caron, Department of Endocrinology and Metabolic Diseases, Hôpital Larrey, 24 Chemin de Pouvourville, 31059 Toulouse Cedex, France; Email: [caron.p@chu-toulouse.fr](mailto:caron.p@chu-toulouse.fr)

**Online Resource 1:** ACRO-POLIS study CRF (English translation)


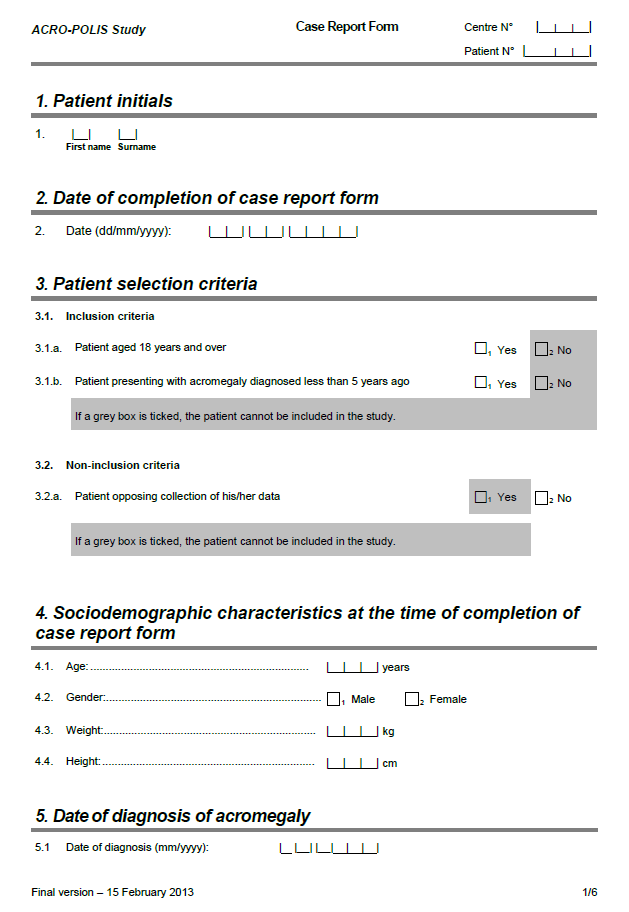


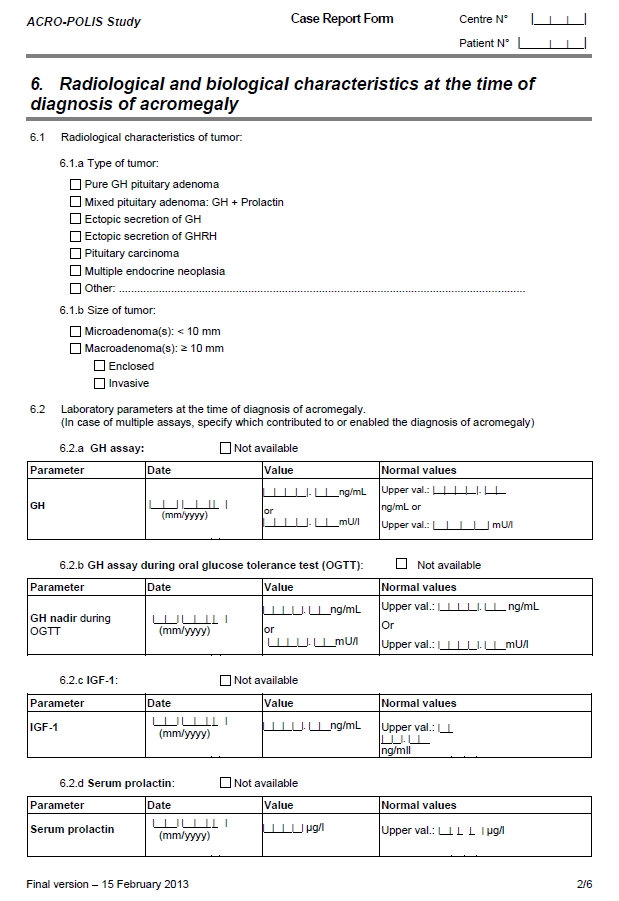


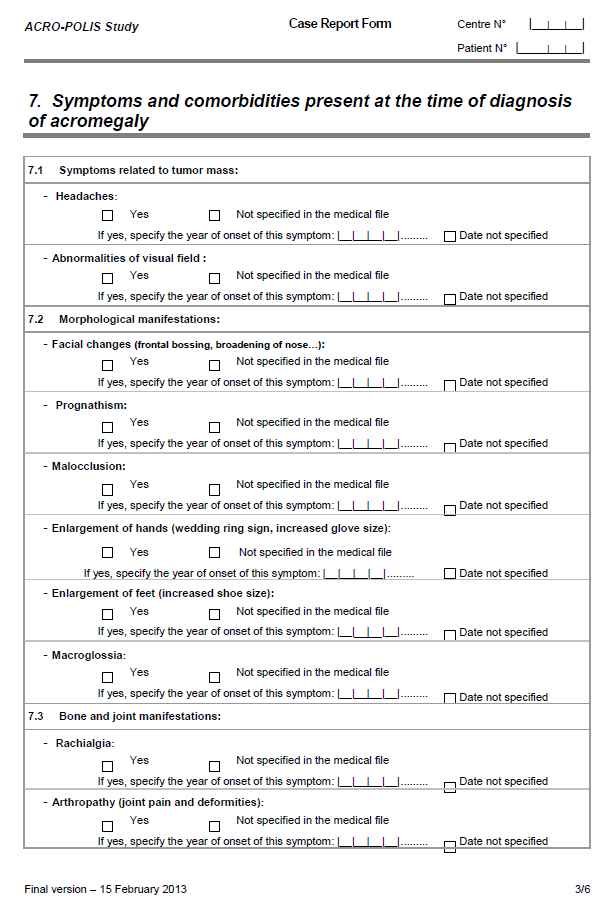


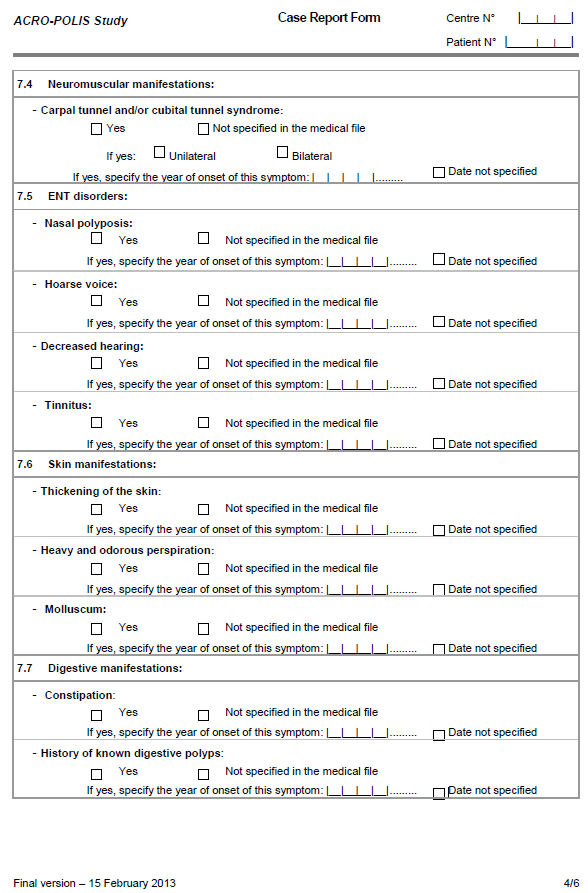


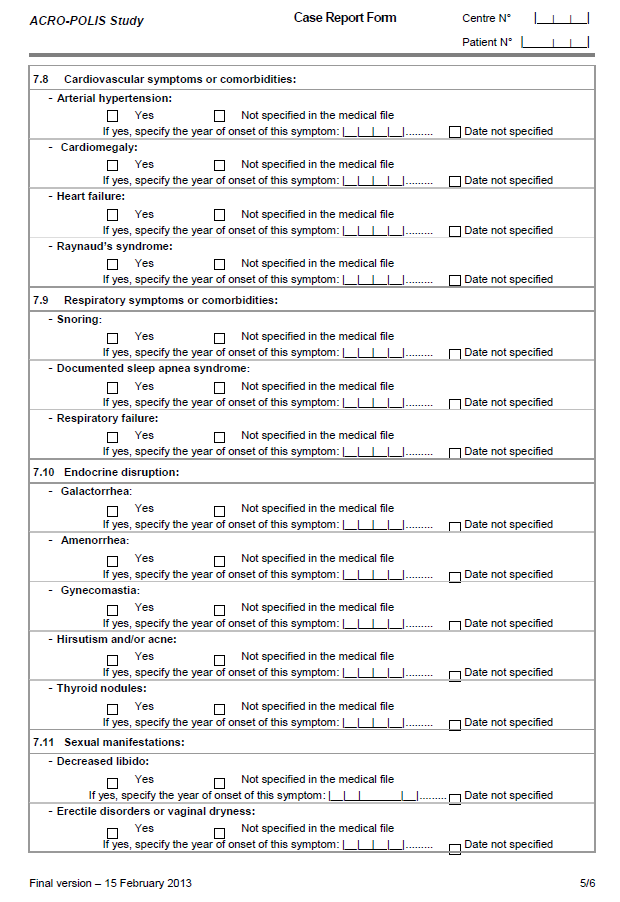


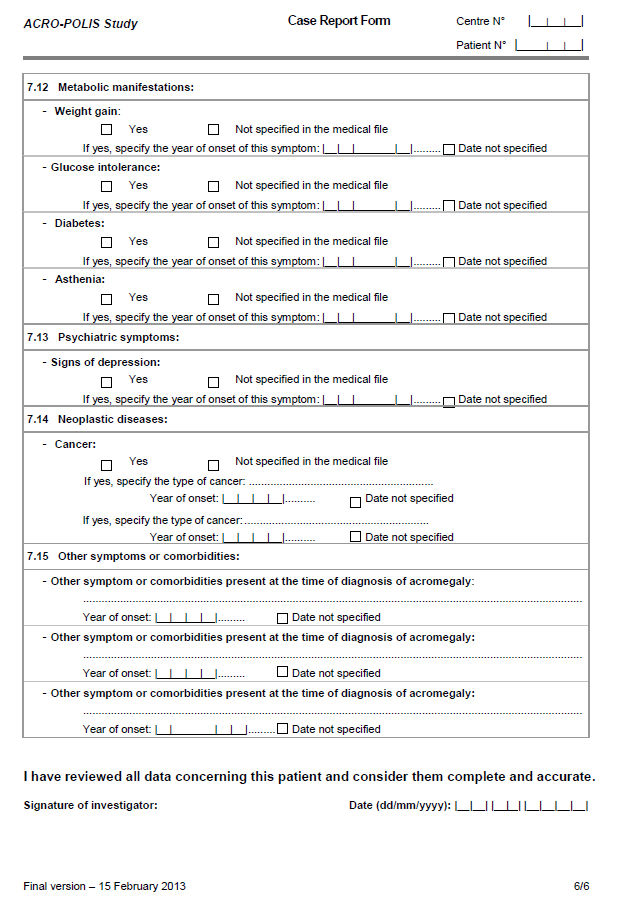


CRF, case report form; GH, growth hormone; GHRH, growth hormone-releasing hormone; IGF-1, insulin-like growth factor; OGTT, oral glucose tolerance test.

**Online Resource 2:** ACRO-POLIS study patient questionnaire (English translation)


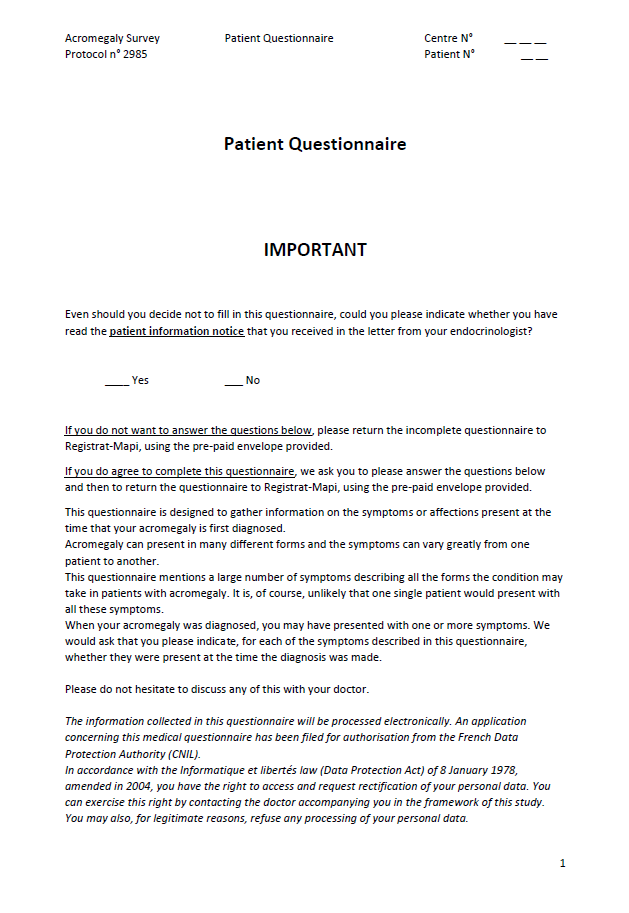


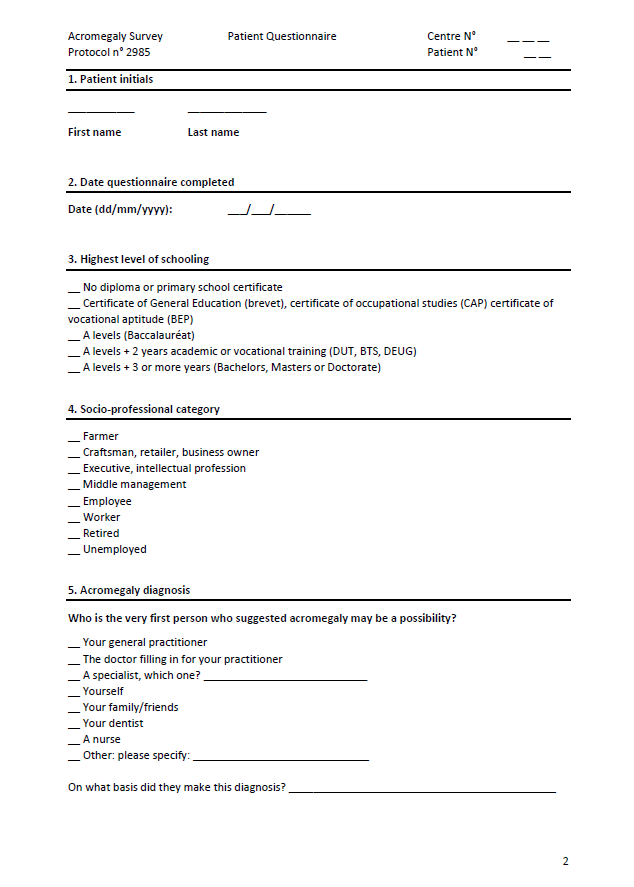


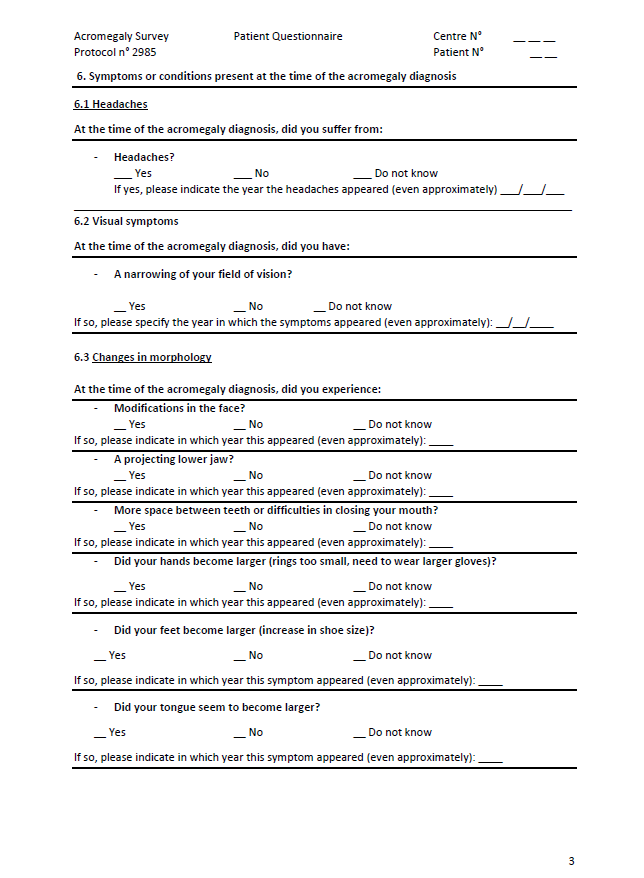


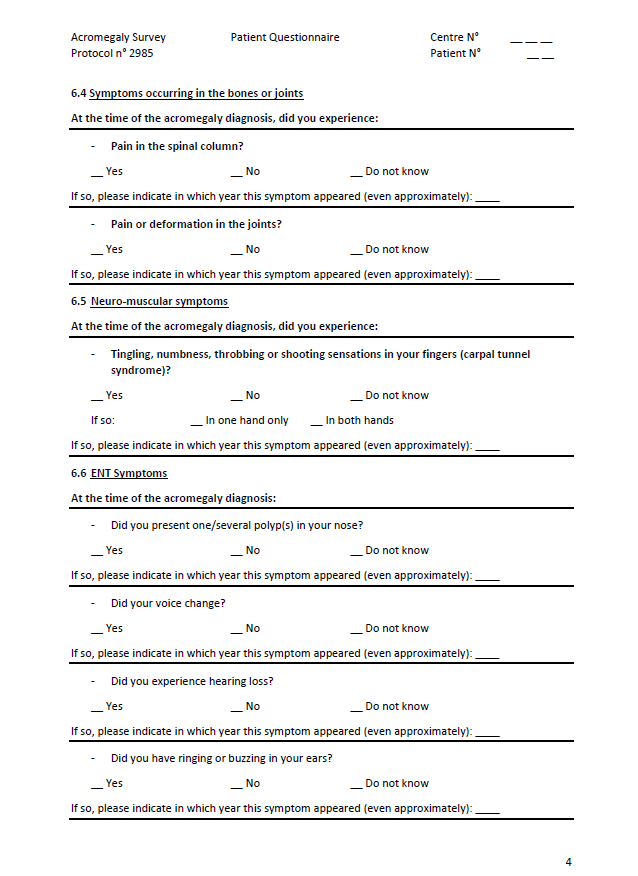


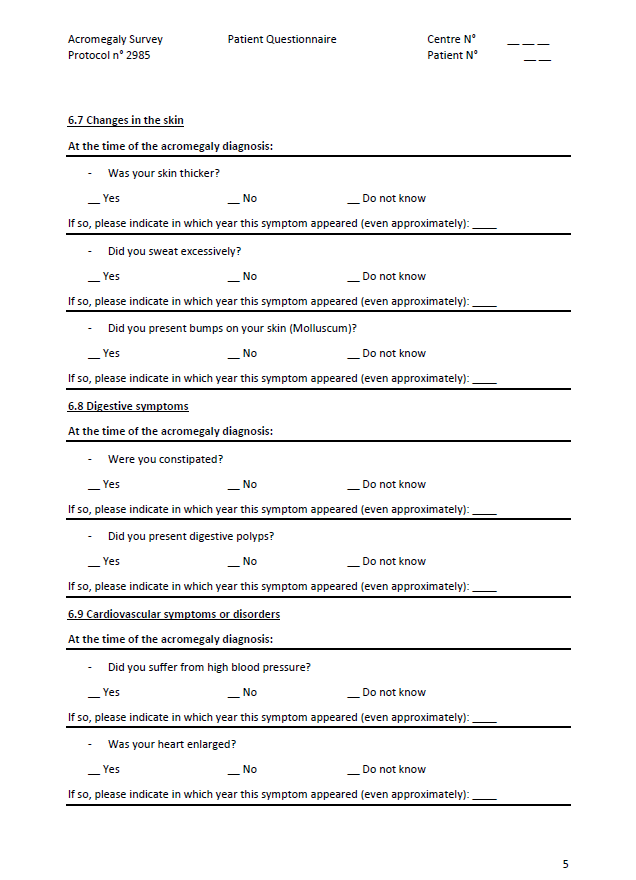


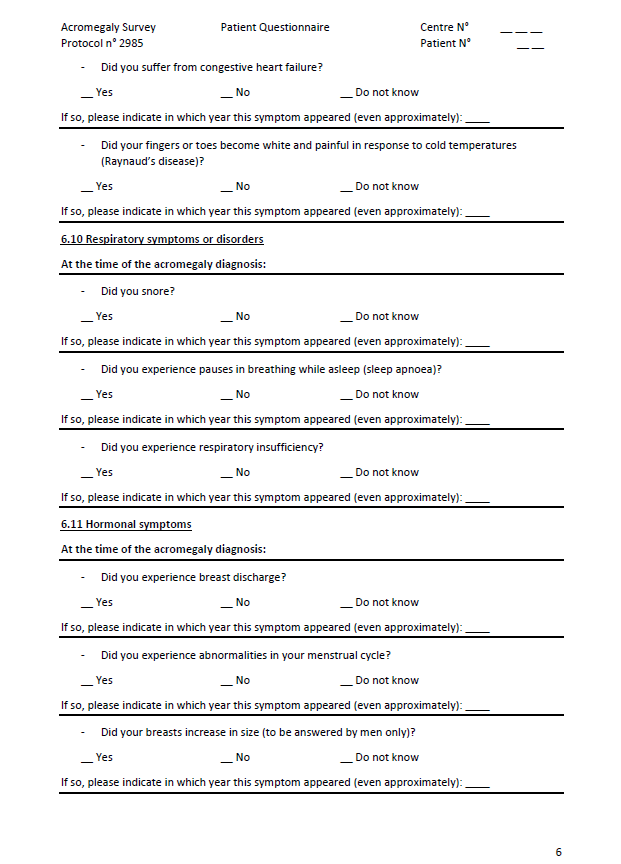


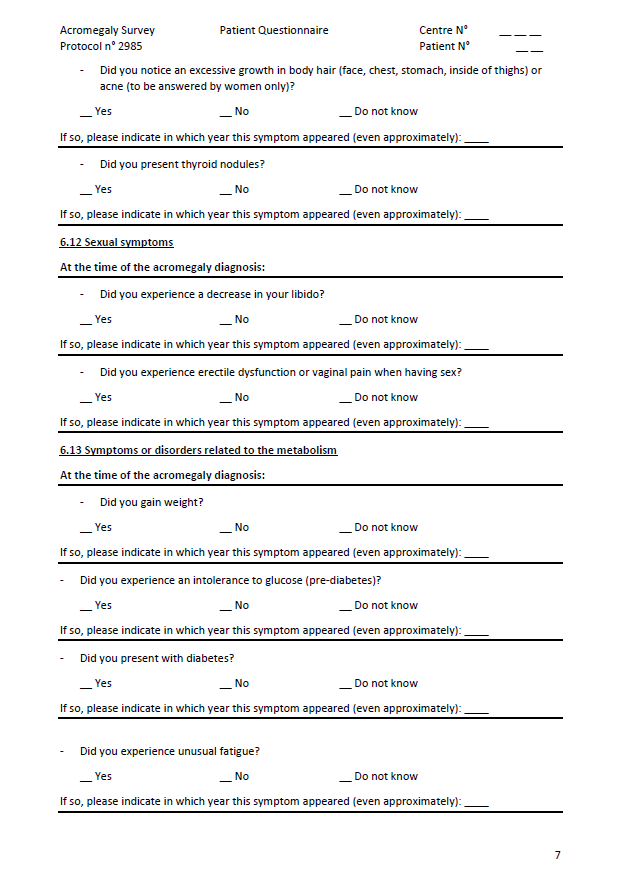


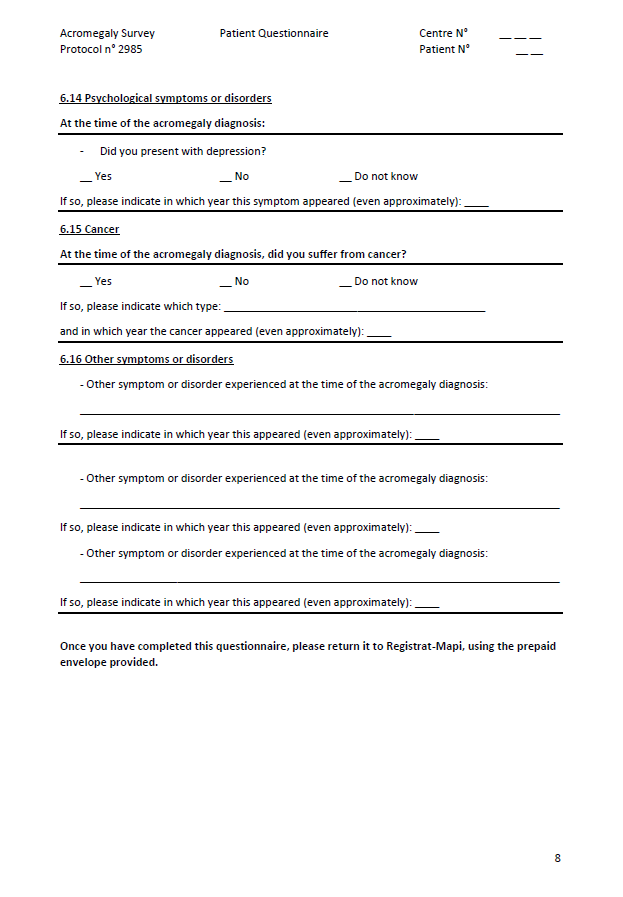


**Online Resource 3.** Management of inconsistencies in the CRF and patient questionnaire in the MCA

| **Symptom or comorbidity in:** | |  |
| --- | --- | --- |
| **CRF** | **Patient-reported questionnaire** | **Decision** |
| “Non-reported in the subject  medical file” or missing data | “Yes” | Yes |
| “Yes” | “Unknown” or missing data | Yes |
| “Yes” | “No” | Yes |
| Date of symptom onset X | Date of symptom onset Y | Date of symptom onset X will be kept |

CRF, case report form; MCA, multiple correspondence analysis.
